# Supplementary material for: Conformational and migrational dynamics of slipped-strand DNA three-way junctions containing trinucleotide repeats
Source: Nat Commun. 2021 Jan 8;12:204. doi: 10.1038/s41467-020-20426-3 (PMC7794359; doi:10.1038/s41467-020-20426-3)
Supplement: Supplementary file 1 — Supplementary Information [file 41467_2020_20426_MOESM1_ESM.pdf]

Supplementary Information for:

Conformational and migrational dynamics of slipped-strand DNA three-way junctions containing trinucleotide repeats

Tianyu Hu, Michael J. Morten, and Steven W. Magennis*

School of Chemistry, University of Glasgow, Joseph Black Building, University Avenue, Glasgow, G12 8QQ, UK

*E-mail: steven.magennis@glasgow.ac.uk

SUPPLEMENTARY TABLES 1-8 pages 2-9

SUPPLEMENTARY FIGURES 1-22 pages 10-31

SUPPLEMENTARY TABLES

Supplementary Table 1. Oligonucleotide sequences for static 3WJs. Labelling positions are indicated by X (C-Cy5) or Y (T-Alexa488).

Name	Oligonucleotide Sequences
Static 1	5' AGT GGT CAG ACG CTG CTG CTG CTG CTG CTG CTG CTG CTG **X**TG CTC GGC ACT CGT GAT TTG GTC
Static 2	5' AGT GGT CAG ACG ATG CTG CTG CTG CTG CTG CTG CTG CTG **X**TG CTG CTC ACT CGT GAT TTG GTC
Static 3	5' AGT GGT CAG ACG ACT ATG CTG CTG CTG CTG CTG CTG CTG **X**TG CTG CTG CTC CGT GAT TTG GTC
Static 4	5' AGT GGT CAG ACG ACT GGC ATG CTG CTG CTG CTG CTG CTG **X**TG CTG CTG CTG CGT GAT TTG GTC
Static CAG 1	5' AGT GGT CAG ACG CAG CAG CAG CAG CAG CAG CAG CAG CAG **X**AG CTC GGC ACT CGT GAT TTG GTC
Static CAG 2	5' AGT GGT CAG ACG ATG CAG CAG CAG CAG CAG CAG CAG CAG **X**AG CAG CTC ACT CGT GAT TTG GTC
Static CAG 3	5' AGT GGT CAG ACG ACT ATG CAG CAG CAG CAG CAG CAG CAG **X**AG CAG CAG CTC CGT GAT TTG GTC
Static CAG 4	5' AGT GGT CAG ACG ACT GGC ATG CAG CAG CAG CAG CAG CAG **X**AG CAG CAG CAG CGT GAT TTG GTC
Static 3-20	5' AGT GGT CAG ACG ACT ATG CTG CTG CTG CTG CTG CTG CTG CTG CTG CTG CTG CTG CTG CTG CTG CTG CTG **X**TG CTG CTG CTC CGT GAT TTG GTC
Static 3-Fully	5' AGT GGT CAG ACG ACT ATG CTG CTG CTG CTG CTG CAG CAG **X**AG CAG CAG CTC CGT GAT TTG GTC
Static 1c	5' /Biotin/GAC CAA ATC ACG AGT GCC GAG CGT C**Y**G ACC ACT
Static 2c	5'/ Biotin/GAC CAA ATC ACG AGT GAG CAT CGT C**Y**G ACC ACT
Static 3c	5' /Biotin/GAC CAA ATC ACG GAG CAT AGT CGT C**Y**G ACC ACT
Static 4c	5' /Biotin/GAC CAA ATC ACG CAT GCC AGT CGT C**Y**G ACC ACT

Supplementary Table 2. Oligonucleotide sequences for mobile 3WJs. Labelling positions are indicated by X (C-Cy5) or Y (T-Alexa488) or Z (C-Atto647N).

Name	Oligonucleotide Sequences
CAG-2	5' AGT GGT CAG ACG CAG **X**AG CAG CAG CAG CGT GAT TTG GTC
CAG-4	5' AGT GGT CAG ACG CAG CAG CAG **X**AG CAG CAG CAG CGT GAT TTG GTC
CAG-6	5' AGT GGT CAG ACG CAG CAG CAG CAG CAG **X**AG CAG CAG CAG CGT GAT TTG GTC
CAG-8	5' AGT GGT CAG ACG CAG CAG CAG CAG CAG CAG CAG **X**AG CAG CAG CAG CGT GAT TTG GTC
CAG-10	5' AGT GGT CAG ACG CAG CAG CAG CAG CAG CAG CAG CAG CAG **X**AG CAG CAG CAG CGT GAT TTG GTC
CAG-20	5' AGT GGT CAG ACG CAG CAG CAG CAG CAG CAG CAG CAG CAG CAG CAG CAG CAG CAG CAG CAG CAG CAG CAG **X**AG CAG CAG CAG CGT GAT TTG GTC
CAG-30	5' AGT GGT CAG ACG CAG CAG CAG CAG CAG CAG CAG CAG CAG CAG CAG CAG CAG CAG CAG CAG CAG CAG CAG CAG CAG CAG CAG CAG CAG CAG CAG CAG CAG **X**AG CAG CAG CAG CGT GAT TTG GTC
CTG-2	5' AGT GGT CAG ACG CTG **X**TG CTG CTG CTG CGT GAT TTG GTC
CTG-4	5' AGT GGT CAG ACG CTG CTG CTG **X**TG CTG CTG CTG CGT GAT TTG GTC
CTG-6	5' AGT GGT CAG ACG CTG CTG CTG CTG CTG **X**TG CTG CTG CTG CGT GAT TTG GTC
CTG-8	5' AGT GGT CAG ACG CTG CTG CTG CTG CTG CTG CTG **X**TG CTG CTG CTG CGT GAT TTG GTC
CTG-10	5' AGT GGT CAG ACG CTG CTG CTG CTG CTG CTG CTG CTG CTG **X**TG CTG CTG CTG CGT GAT TTG GTC
CTG-20	5' AGT GGT CAG ACG CTG CTG CTG CTG CTG CTG CTG CTG CTG CTG CTG CTG CTG CTG CTG CTG CTG CTG CTG **X**TG CTG CTG CTG CGT GAT TTG GTC
CTG-30	5' AGT GGT CAG ACG CTG CTG CTG CTG CTG CTG CTG CTG CTG CTG CTG CTG CTG CTG CTG CTG CTG CTG CTG CTG CTG CTG CTG CTG CTG CTG CTG CTG CTG **X**TG CTG CTG CTG CGT GAT TTG GTC
Atto	5' AGT GGT CAG ACG CTG CTG CTG CTG CTG CTG CTG CTG CTG **Z**TG CTG CTG CTG CGT GAT TTG GTC
DP	5' AGT GGT CAG ACG CTG CTG CTG CTG CTG CTG CTG CTG CTG CTG **X**TG CTG CTG CGT GAT TTG GTC
CAG_comp	5' /Biotin/ GAC CAA ATC ACG CTG CTG CTG CGT C**Y**G ACC ACT
CTG_comp	5' /Biotin/ GAC CAA ATC ACG CAG CAG CAG CGT C**Y**G ACC ACT

Supplementary Table 3. Strands used to form static 3WJs.

3WJs	Strands
S1	Static 1, Static 1c
S2	Static 2, Static 2c
S3	Static 3, Static 3c
S4	Static 4, Static 4c
S-CAG-1	Static CAG 1, Static 1c
S-CAG-2	Static CAG 2, Static 2c
S-CAG-3	Static CAG 3, Static 3c
S-CAG-4	Static CAG 4, Static 4c
S3_20	Static 3-20, Static 3c
S3_F	Static 3-Fully, Static 3c

Supplementary Table 4. Strands used to form mobile 3WJs.

3WJs	Strands
$(CAG)_2$	CAG-2, CAG_comp
$(CAG)_4$	CAG-4, CAG_comp
$(CAG)_6$	CAG-6, CAG_comp
$(CAG)_8$	CAG-8, CAG_comp
$(CAG)_{10}$	CAG-10, CAG_comp
$(CAG)_{20}$	CAG-20, CAG_comp
$(CAG)_{30}$	CAG-30, CAG_comp
$(CTG)_2$	CTG-2, CTG_comp
$(CTG)_4$	CTG-4, CTG_comp
$(CTG)_6$	CTG-6, CTG_comp
$(CTG)_8$	CTG-8, CTG_comp
$(CTG)_{10}$	CTG-10, CTG_comp
$(CTG)_{20}$	CTG-20, CTG_comp
$(CTG)_{30}$	CTG-30, CTG_comp
$(CTG)_{10}$-Atto	Atto, CTG_comp
$(CTG)_{10}$-DP	DP, CTG_comp

Supplementary Table 5. FRET levels for static and mobile 3WJs calculated by MFD and TIRF.[a]

FRET State	1		2		3		4		5		6	
Method	MFD	TIRF	MFD	TIRF	MFD	TIRF	MFD	TIRF	MFD	TIRF	MFD	TIRF
S1	0.69 (0.31)	0.68 (0.22)	0.87 (0.12)	0.87 (0.22)								
S2	0.54 (0.54)	0.53 (0.20)	0.80 (0.12)	0.75 (0.20)								
S3	0.12 (0.19)	0.11 (0.22)	0.53 (0.26)	0.40 (0.22)								
S4	0.09 (0.13)											
S-CAG-1	0.75 (0.25)	0.66 (0.28)	0.87 (0.09)	0.87 (0.28)								
S-CAG-2	0.42 (0.71)	0.47 (0.34)	0.75 (0.14)	0.78 (0.34)								
S-CAG-3	0.07 (0.13)	0.10 (0.26)	0.43 (0.37)	0.38 (0.26)								
S-CAG-4	0.05 (0.09)	0.08 (0.20)	0.21 (0.32)	0.31 (0.20)								
$(CAG)_2$	0.70 (0.29)											
$(CAG)_4$	0.30 (0.23)	0.28 (0.36)	0.86 (0.13)	0.84 (0.36)								
$(CAG)_6$	0.02 (0.04)	0.11 (0.24)	0.10 (0.18)	0.35 (0.24)	0.53 (0.53)	0.49 (0.24)	0.81 (0.13)	0.61 (0.24)	0.87 (0.07)	0.82 (0.24)	0.90 (0.04)	0.92 (0.24)
$(CAG)_8$	0.01 (0.04)	0.07 (0.22)	0.05 (0.09)	0.24 (0.22)	0.45 (0.69)	0.37 (0.22)	0.82 (0.13)	0.53 (0.22)	0.88 (0.06)	0.77 (0.22)	0.91 (0.04)	0.91 (0.22)
$(CAG)_{10}$	0.03 (0.06)	0.06 (0.20)	0.09 (0.12)	0.25 (0.20)	0.51 (0.47)	0.42 (0.20)	0.78 (0.11)	0.59 (0.20)	0.86 (0.08)	0.79 (0.20)	0.92 (0.05)	0.92 (0.20)
$(CAG)_{20}$	0.02 (0.05)	0.08 (0.22)	0.08 (0.11)	0.23 (0.22)	0.47 (0.53)	0.44 (0.22)	0.80 (0.14)	0.55 (0.22)	0.86 (0.08)	0.75 (0.22)	0.92 (0.04)	0.91 (0.22)
$(CAG)_{30}$	0.02 (0.05)	0.09 (0.20)	0.06 (0.11)	0.25 (0.20)	0.43 (0.65)	0.43 (0.20)	0.81 (0.15)	0.60 (0.20)	0.89 (0.07)	0.80 (0.20)	0.92 (0.02)	0.92 (0.20)
$(CTG)_2$	0.69 (0.30)											
$(CTG)_4$	0.31 (0.23)	0.22 (0.34)	0.85 (0.11)	0.84 (0.34)								
$(CTG)_6$	0.02 (0.05)	0.10 (0.26)	0.14 (0.29)	0.28 (0.26)	0.66 (0.34)	0.34 (0.26)	0.83 (0.11)	0.50 (0.26)	0.87 (0.05)	0.73 (0.26)	0.90 (0.04)	0.91 (0.26)
$(CTG)_8$	0.02 (0.04)	0.08 (0.22)	0.12 (0.12)	0.20 (0.22)	0.60 (0.46)	0.41 (0.22)	0.84 (0.12)	0.53 (0.22)	0.90 (0.06)	0.73 (0.22)	0.92 (0.02)	0.91 (0.22)
$(CTG)_{10}$	0.15 (0.13)											
$(CTG)_{20}$	0.02 (0.04)	0.09 (0.26)	0.09 (0.15)	0.23 (0.26)	0.57 (0.46)	0.41 (0.26)	0.82 (0.11)	0.61 (0.26)	0.87 (0.07)	0.80 (0.26)	0.92 (0.05)	0.90 (0.26)
$(CTG)_{30}$	0.02 (0.04)	0.10 (0.24)	0.08 (0.14)	0.26 (0.24)	0.57 (0.51)	0.40 (0.24)	0.82 (0.13)	0.59 (0.24)	0.87 (0.08)	0.77 (0.24)	0.92 (0.04)	0.90 (0.24)

[a] For MFD, FRET states were obtained from Gaussian fits of the FRET histogram. For TIRF, FRET states were obtained from Hidden Markov Modelling. Full width half maxima are shown in parentheses.

Supplementary Table 6. FRET levels calculated by MFD and TIRF for a static 3WJ with 20 CTG repeats (S3_20), with a fully-complementary slip-out of 10 CTG repeats (S3_F), with Atto647N as the acceptor dye [$(CTG)_{10}$-Atto)] and with an alternate location for Cy5 [$(CTG)_{10}$-DP].[a]

FRET State	1		2		3		4		5		6	
Method	MFD	TIRF	MFD	TIRF	MFD	TIRF	MFD	TIRF	MFD	TIRF	MFD	TIRF
S3_20	0.14 (0.21)	0.13 (0.32)	0.55 (0.24)	0.48 (0.32)								
S3_F	0.15 (0.16)	0.09 (0.18)	0.53 (0.24)	0.34 (0.18)								
$(CTG)_{10}$-Atto	0.04 (0.07)	0.06 (0.22)	0.28 (0.47)	0.23 (0.22)	0.71 (0.20)	0.39 (0.22)	0.81 (0.11)	0.62 (0.22)	0.87 (0.07)	0.81 (0.22)	0.92 (0.04)	0.90 (0.22)
$(CTG)_{10}$-DP	0.07 (0.11)		0.29 (0.32)		0.49 (0.07)		0.66 (0.17)		0.79 (0.09)		0.84 (0.08)	

[a] For MFD, FRET states were obtained from Gaussian fits of the FRET histogram. For TIRF, FRET states were obtained from Hidden Markov Modelling. Full width half maxima are shown in parentheses.

Supplementary Table 7. Fractional occupancy[a] of FRET states for mobile 3WJs with CAG slip-outs.

$(CAG)_6$		$(CAG)_8$		$(CAG)_{10}$		$(CAG)_{20}$		$(CAG)_{30}$	
Transition	Fraction Spent	Transition	Fraction Spent	Transition	Fraction Spent	Transition	Fraction Spent	Transition	Fraction Spent
6→3	0.3623	6→4	0.2323	1→3	0.2087	6→3	0.2381	6→3	0.2613
5→3	0.1427	2→1	0.0841	6→3	0.1829	6→5	0.1066	1→3	0.1067
1→3	0.0739	3→2	0.0738	5→3	0.0766	6→1	0.0954	2→4	0.0641
2→4	0.0563	5→4	0.0594	6→4	0.0738	1→3	0.0844	5→3	0.0641
6→1	0.0552	1→2	0.0480	6→1	0.0608	5→6	0.0429	6→1	0.0614
6→2	0.0412	2→4	0.0466	1→2	0.0434	1→6	0.0360	4→2	0.0577
4→2	0.0345	5→6	0.0426	6→5	0.0390	1→5	0.0358	1→6	0.0454
4→1	0.0321	6→1	0.0363	1→6	0.0386	1→2	0.0353	6→5	0.0437
2→1	0.0308	3→6	0.0361	1→5	0.0282	6→4	0.0303	1→4	0.0338
3→6	0.0306	1→4	0.0358	2→1	0.0258	3→6	0.0280	1→5	0.0315
5→4	0.0282	6→5	0.0356	3→1	0.0257	3→1	0.0274	5→4	0.0311
1→2	0.0243	2→3	0.0352	4→3	0.0237	2→3	0.0268	3→5	0.0297
4→5	0.0205	1→3	0.0233	4→6	0.0211	4→2	0.0266	1→2	0.0225
1→6	0.0177	6→3	0.0233	3→6	0.0208	5→3	0.0253	6→4	0.0191
3→1	0.0159	4→6	0.0232	3→5	0.0196	2→1	0.0204	3→1	0.0165
3→5	0.0118	4→5	0.0230	5→1	0.0162	6→2	0.0203	2→3	0.0162
1→4	0.0068	4→1	0.0190	2→4	0.0155	3→5	0.0153	3→6	0.0141
5→1	0.0068	6→2	0.0163	2→3	0.0121	5→1	0.0152	2→1	0.0116
2→6	0.0063	1→6	0.0151	5→6	0.0101	4→6	0.0123	4→5	0.0110
2→5	0.0009	2→5	0.0146	3→2	0.0089	3→2	0.0120	5→2	0.0106
1→5	0.0000	5→2	0.0139	4→2	0.0088	2→4	0.0119	5→6	0.0096
2→3	0.0000	4→3	0.0133	3→4	0.0079	4→5	0.0115	2→5	0.0088
3→2	0.0000	4→2	0.0116	5→4	0.0070	5→4	0.0090	6→2	0.0074
3→4	0.0000	5→3	0.0109	4→5	0.0055	3→4	0.0083	4→6	0.0064
4→3	0.0000	3→5	0.0109	2→6	0.0053	4→3	0.0082	5→1	0.0060
4→6	0.0000	3→4	0.0075	4→1	0.0046	2→5	0.0067	4→1	0.0043
5→2	0.0000	3→1	0.0072	5→2	0.0041	5→2	0.0045	3→2	0.0036
5→6	0.0000	5→1	0.0008	6→2	0.0024	2→6	0.0032	3→4	0.0012
6→4	0.0000	1→5	0.0000	1→4	0.0019	1→4	0.0022	2→6	0.0000
6→5	0.0000	2→6	0.0000	2→5	0.0011	4→1	0.0000	4→3	0.0000

[a] Fraction of the entire FRET trajectory that is spent in a start FRET state that eventually transits into an end FRET state.

Supplementary Table 8. Fractional occupancy[a] of FRET states for mobile 3WJs with CTG slip-outs.

$(CTG)_6$		$(CTG)_8$		$(CTG)_{20}$		$(CTG)_{30}$	
Transition	Fraction Spent	Transition	Fraction Spent	Transition	Fraction Spent	Transition	Fraction Spent
6→3	0.3908	6→3	0.2158	6→1	0.1674	6→1	0.1566
5→3	0.0891	1→3	0.2109	6→3	0.0951	6→4	0.0889
6→1	0.0860	6→1	0.1043	5→3	0.0809	1→6	0.0874
1→5	0.0652	1→6	0.0797	1→4	0.0685	6→5	0.0610
2→1	0.0514	2→4	0.0452	6→4	0.0531	2→1	0.0598
3→6	0.0338	6→5	0.0358	3→5	0.0529	1→2	0.0588
6→4	0.0338	2→1	0.0344	1→6	0.0456	1→3	0.0567
1→2	0.0299	6→2	0.0285	4→1	0.0431	5→3	0.0538
2→4	0.0256	1→5	0.0247	6→5	0.0378	1→4	0.0446
2→6	0.0256	2→5	0.0239	6→2	0.0367	5→6	0.0372
1→6	0.0216	3→6	0.0234	2→3	0.0327	3→1	0.0342
6→2	0.0180	1→4	0.0225	1→3	0.0306	3→5	0.0292
4→2	0.0172	5→6	0.0192	5→1	0.0292	5→2	0.0285
4→1	0.0170	4→2	0.0191	1→5	0.0254	6→3	0.0226
5→2	0.0149	3→1	0.0170	2→6	0.0224	2→4	0.0200
1→4	0.0127	5→3	0.0144	2→1	0.0222	5→4	0.0190
3→5	0.0109	5→2	0.0133	4→6	0.0219	4→6	0.0170
4→5	0.0108	5→1	0.0108	1→2	0.0218	4→5	0.0168
6→5	0.0107	4→5	0.0106	3→1	0.0173	4→2	0.0153
5→1	0.0095	2→6	0.0096	5→4	0.0156	5→1	0.0120
4→6	0.0089	6→4	0.0091	4→3	0.0144	2→5	0.0108
5→4	0.0077	1→2	0.0085	2→4	0.0127	3→2	0.0106
2→5	0.0046	4→6	0.0085	5→6	0.0122	3→4	0.0105
1→3	0.0000	3→2	0.0065	3→2	0.0114	4→3	0.0102
2→3	0.0000	5→4	0.0020	3→4	0.0104	6→2	0.0099
3→1	0.0000	3→5	0.0016	4→2	0.0085	1→5	0.0086
3→2	0.0000	3→4	0.0005	3→6	0.0063	2→3	0.0084
3→4	0.0000	4→3	0.0003	4→5	0.0014	4→1	0.0073
4→3	0.0000	2→3	0.0000	2→5	0.0000	3→6	0.0035
5→6	0.0000	4→1	0.0000	5→2	0.0000	2→6	0.0004

[a] Fraction of the entire FRET trajectory that is spent in a start FRET state that eventually transits into an end FRET state.

SUPPLEMENTARY FIGURES

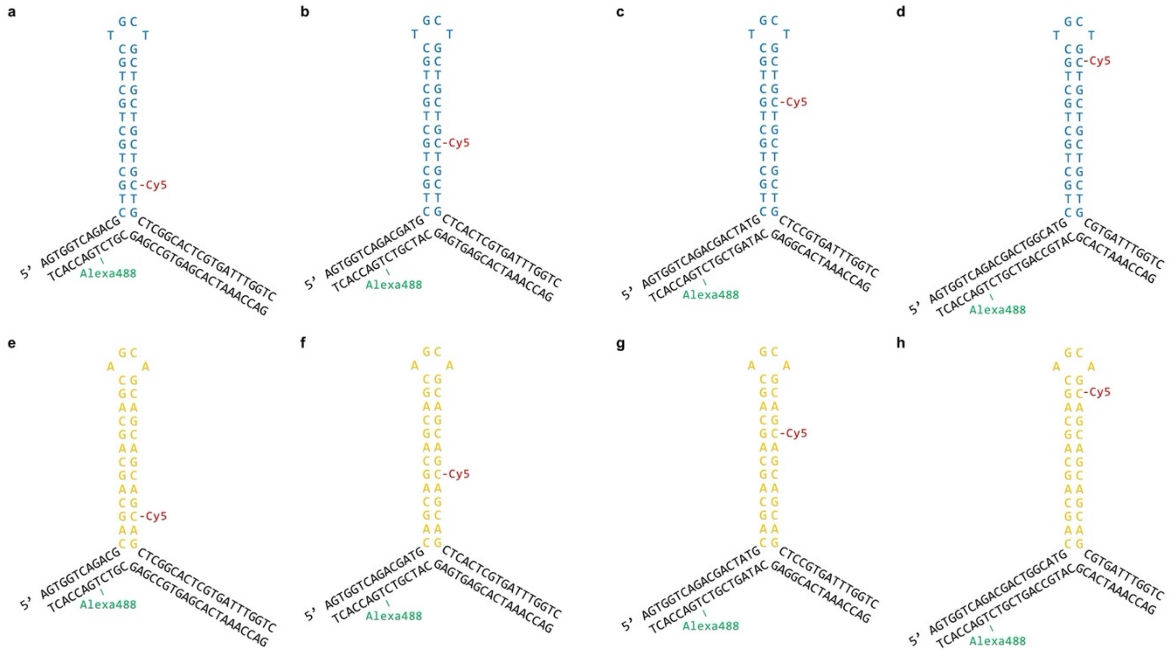

Supplementary Fig. 1. Structure of static 3WJs with $(CTG)_{10}$ and $(CAG)_{10}$ slip-outs. Static 3WJs formed from hybridisation of two DNA strands. One strand is labelled with Alexa488 and the other with Cy5. The CTG slipouts are S1 (**a**), S2 (**b**), S3 (**c**) and S4 (**d**); the CAG slipouts are S-CAG-1 (**e**), S-CAG-2 (**f**), S-CAG-3 (**g**) and S-CAG-4 (**h**).

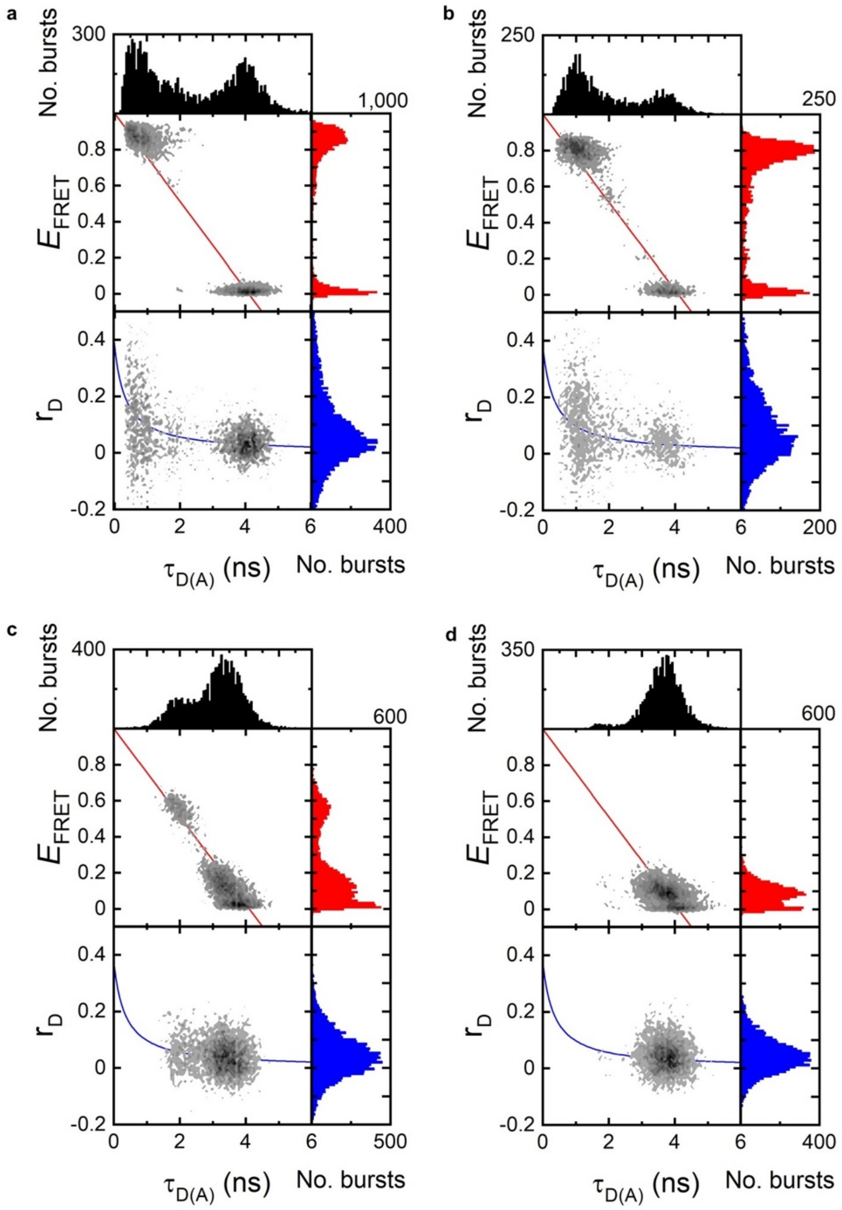

Supplementary Fig. 2. SM-FRET of static (CTG)$_{10}$ 3WJs using MFD: 2D plots. a-d, 2D plot shows FRET efficiency (E_{FRET}) or donor anisotropy (r_D) vs donor lifetime [$\tau_{D(A)}$] for S1 (**a**), S2 (**b**), S3 (**c**) and S4 (**d**). The overlaid red line is the theoretical FRET relationship $E_{FRET} = 1 - [\tau_{D(A)}/\tau_D]$, where τ_D = 4.1 ns. The blue overlaid line is the Perrin equation $r_D = r_0/[1 + \tau_{D(A)}/\rho_D]$, with mean rotational correlation time ρ_D = 0.35 ns and fundamental anisotropy r_0 = 0.375. The gray scale indicates an increasing number of single-molecule bursts from white to black. The samples were measured at 20 ℃ in a buffer with 1 mM $MgCl_2$.

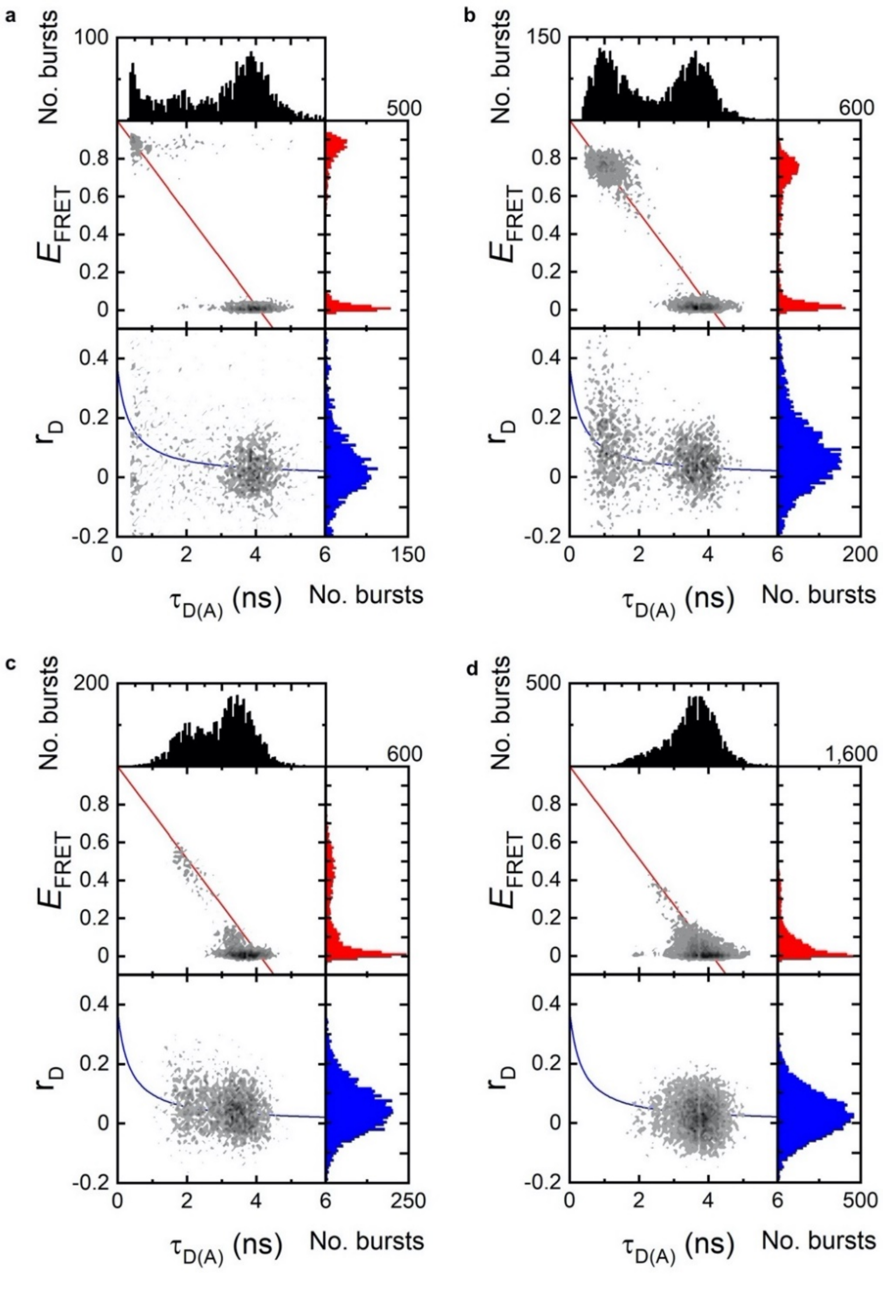

Supplementary Fig. 3. SM-FRET of static (CAG)$_{10}$ 3WJs using MFD: 2D plots. a-d, 2D plot shows FRET efficiency (E_{FRET}) or donor anisotropy (r_D) vs donor lifetime [$\tau_{D(A)}$] for S-CAG-1 (**a**), S-CAG-2 (**b**), S-CAG-3 (**c**) and S-CAG-4 (**d**). The overlaid red line is the theoretical FRET relationship $E_{FRET} = 1 - [\tau_{D(A)}/\tau_D]$, where τ_D = 4.1 ns. The blue overlaid line is the Perrin equation $r_D = r_0/[1 + \tau_{D(A)}/\rho_D]$, with mean rotational correlation time ρ_D = 0.35 ns and fundamental anisotropy r_0 = 0.375. The gray scale indicates an increasing number of single-molecule bursts from white to black. The samples were measured at 20 °C in a buffer with 1 mM $MgCl_2$.

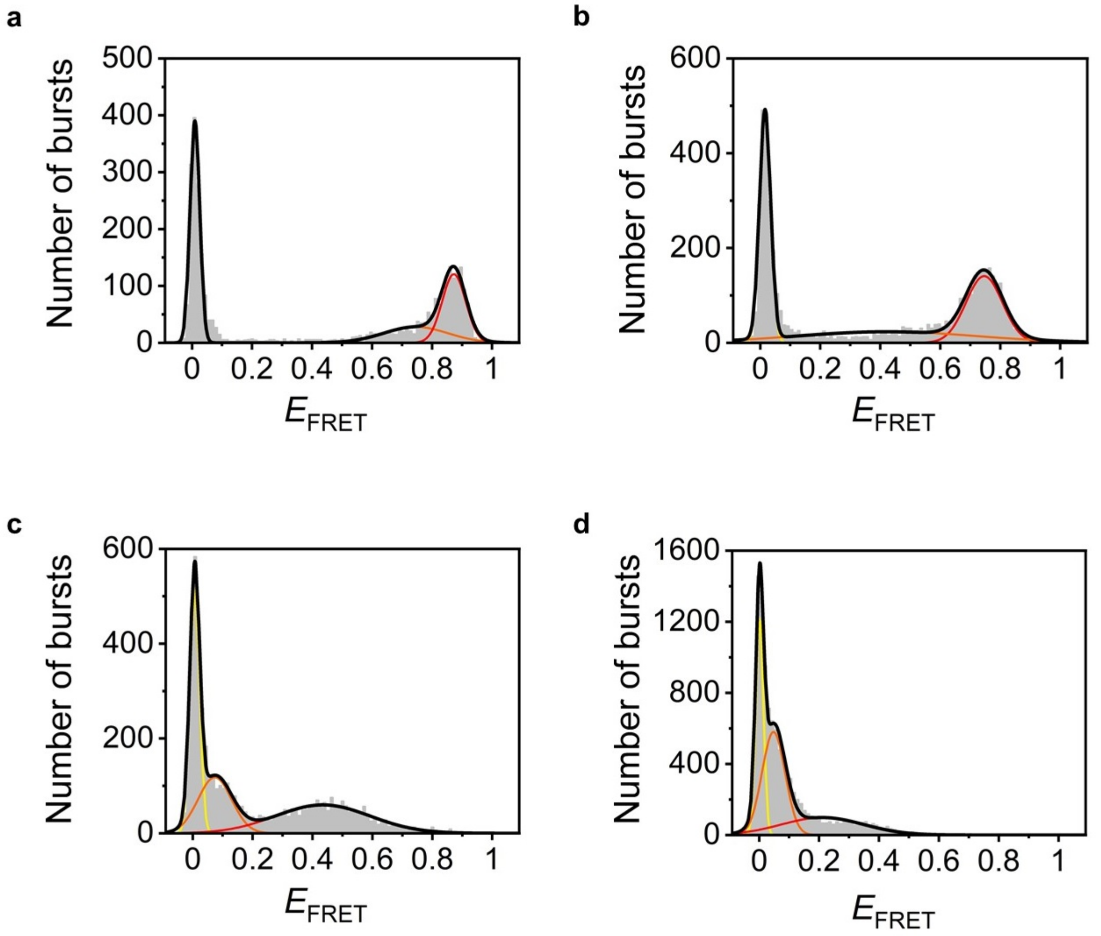

Supplementary Fig. 4. SM-FRET of static $(CAG)_{10}$ 3WJs using MFD: FRET histograms. FRET histograms of FRET efficiency (E_{FRET}) vs. number of bursts for S-CAG-1 (a), S-CAG-2 (b), S-CAG-3 (c), and S-CAG-4 (d) fitted to one donor-only state and two FRET states. The samples were measured at 20 °C in a buffer with 1 mM $MgCl_2$. See Supplementary Fig. 1 for the structures of S-CAG-1-4.

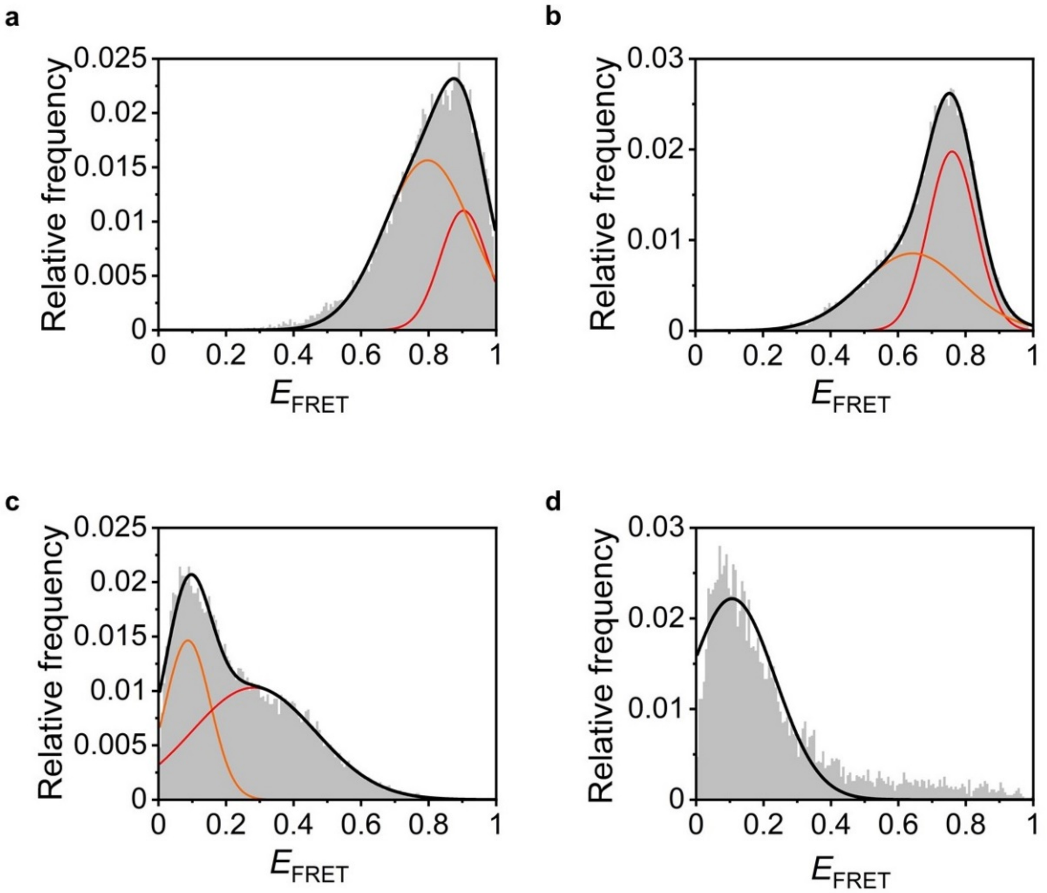

Supplementary Fig. 5. SM-FRET using TIRF for static $(CTG)_{10}$ 3WJs. FRET histograms for S1 (**a**), S2 (**b**) and S3 (**c**) and S4 (**d**) fitted to one (S4) or two Gaussians (S1-S3). The samples were measured at 20 ℃ in a buffer with 1 mM $MgCl_2$. See Supplementary Fig. 1 for the structures of S1-4.

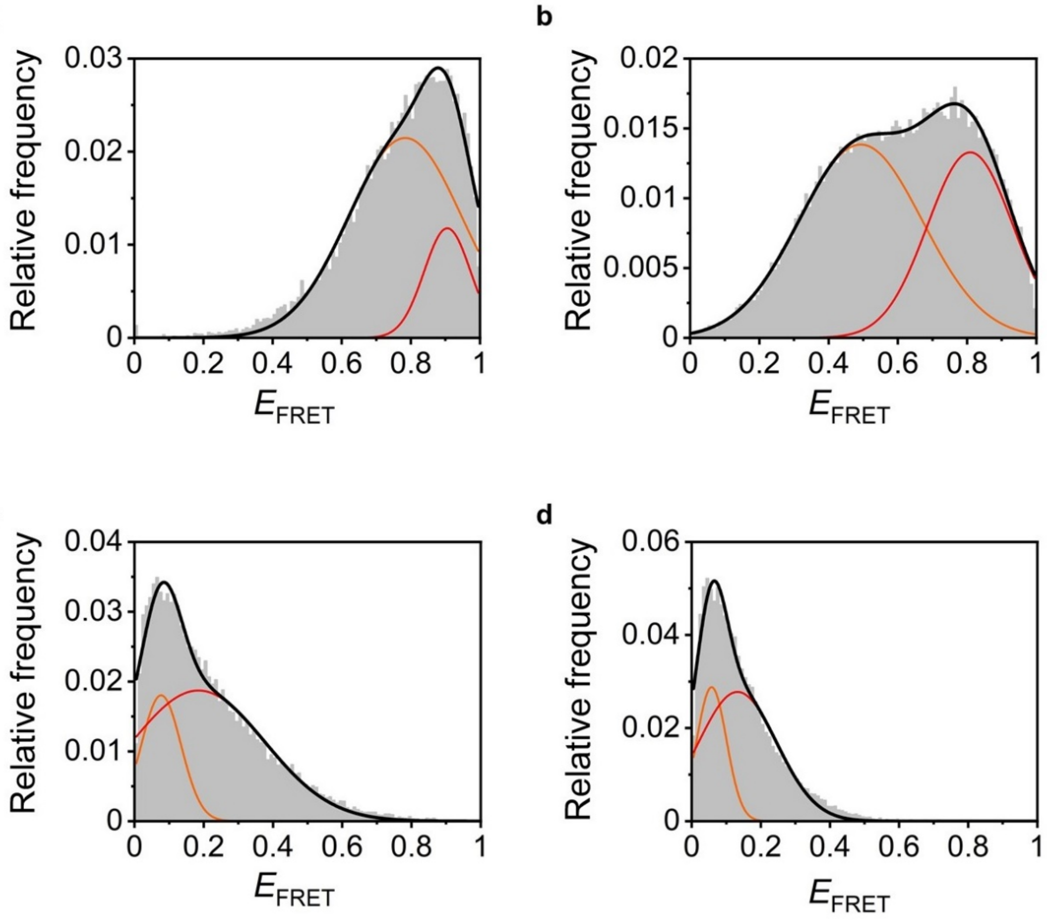

Supplementary Fig. 6. SM-FRET using TIRF for static $(CAG)_{10}$ 3WJs. FRET histograms for S-CAG-1 (**a**), S-CAG-2 (**b**), S-CAG-3 (**c**) and S-CAG-4 (**d**) fitted to two Gaussians. The samples were measured at 20 °C in a buffer with 1 mM $MgCl_2$. See Supplementary Fig. 1 for the structures of S-CAG-1-4.

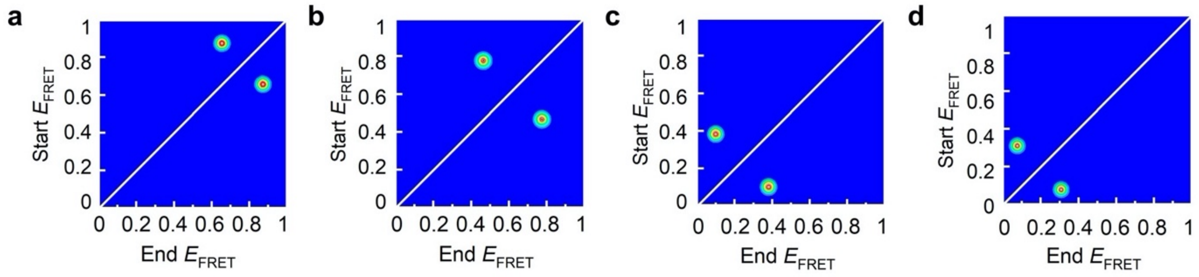

Supplementary Fig. 7. Transition density plots for static $(CAG)_{10}$ 3WJs. Transition density plots from TIRF time traces of static 3WJs with $(CAG)_{10}$ for S-CAG-1 (a), S-CAG-2 (b), S-CAG-3 (c), and S-CAG-4 (d). The samples were measured at 20 °C in a buffer with 1 mM $MgCl_2$. See Supplementary Fig. 1 for the structures of S-CAG-1-4.

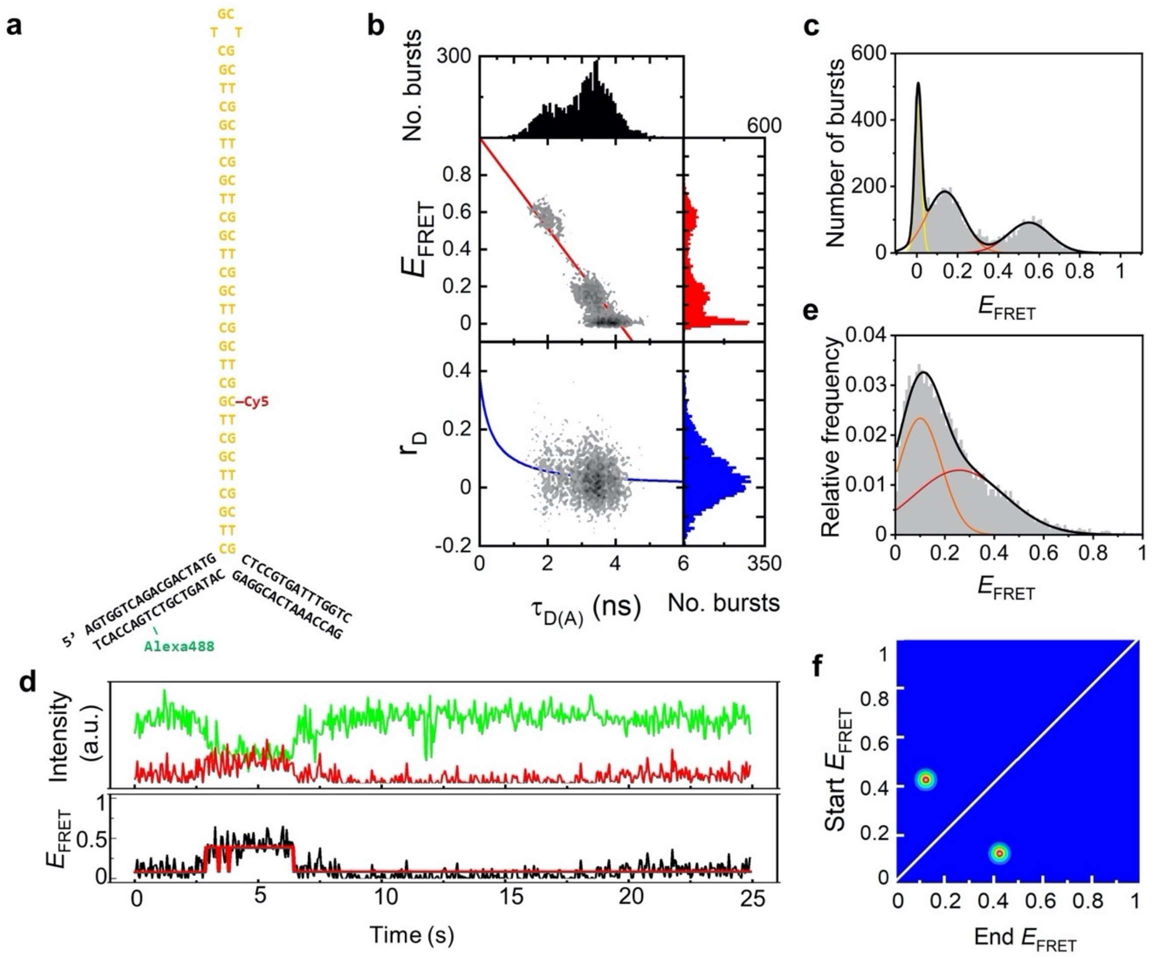

Supplementary Fig. 8. SM-FRET for a static 3WJ with 20 CTG repeats. a, Sequence and dye positions. **b,** 2D plot shows FRET efficiency (E_{FRET}) or donor anisotropy (r_D) vs donor lifetime [$\tau_{D(A)}$] The overlaid red line is the theoretical FRET relationship $E_{FRET} = 1 - [\tau_{D(A)}/\tau_D]$, where τ_D = 4.1 ns. The blue overlaid line is the Perrin equation $r_D = r_0/[1 + \tau_{D(A)}/\rho_D]$, with mean rotational correlation time ρ_D = 0.35 ns and fundamental anisotropy r_0 = 0.375. The gray scale indicates an increasing number of single-molecule bursts from white to black. **c,** FRET histograms from MFD fitted to one donor-only state and two FRET states. **d,** TIRF time traces of surface-immobilized 3WJ; Alexa488 (green) and Cy5 (red) intensities, and calculated FRET efficiency (E_{FRET}) and idealized trace (red) from Hidden Markov modeling. **e,** FRET histograms from TIRF fitted to two FRET states. **f,** Transition density plots of TIRF FRET traces. Measured at 20 °C in a buffer containing 1 mM $MgCl_2$.

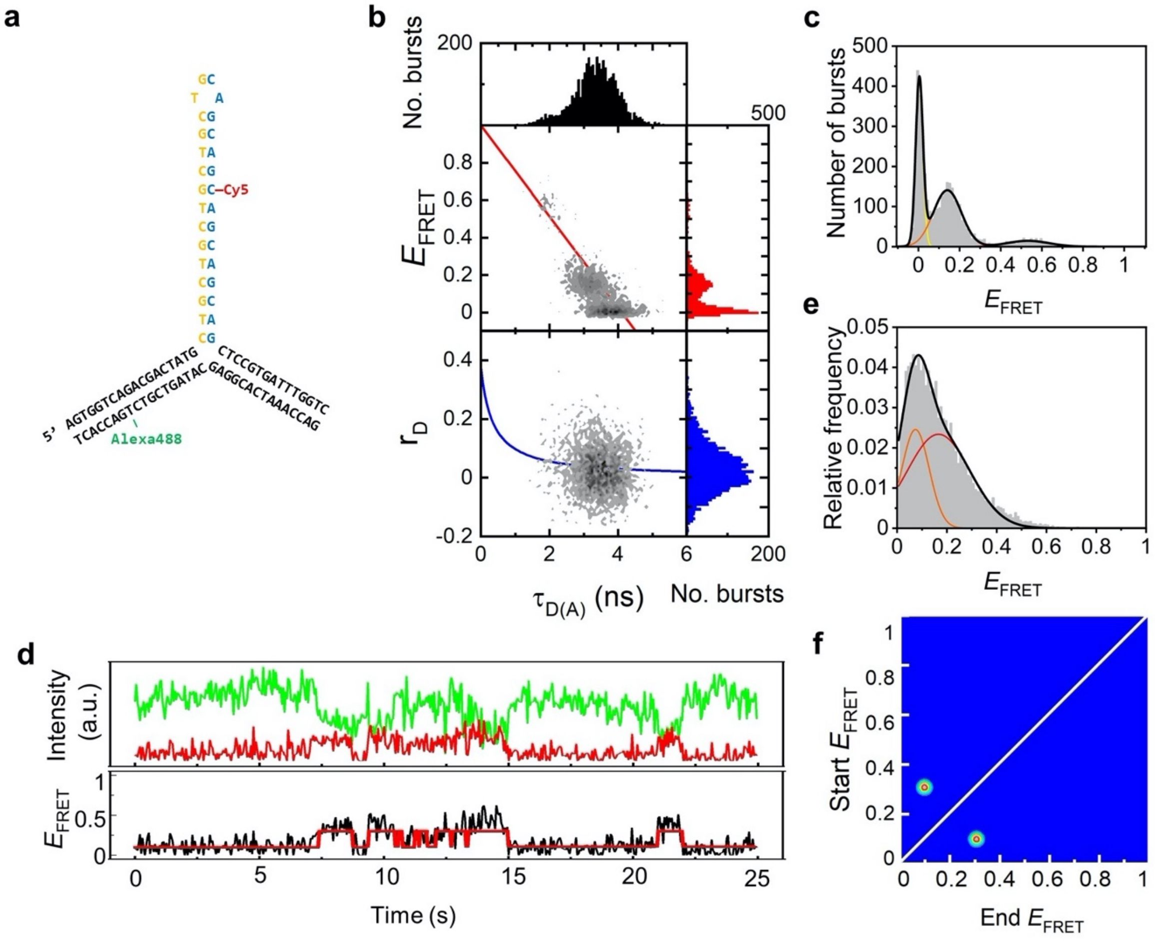

Supplementary Fig. 9. SM-FRET for a fully-complementary 3WJ with a 10-repeat slip-out. a, Sequence and dye positions. **b,** 2D plot shows FRET efficiency (E_{FRET}) or donor anisotropy (r_D) vs donor lifetime [$\tau_{D(A)}$] The overlaid red line is the theoretical FRET relationship $E_{\ FRET} = 1 - \left[\tau_{D(A)}/\tau_D\right]$, where τ_D = 4.1 ns. The blue overlaid line is the Perrin equation $r_D = r_0/\left[1 + \tau_{D(A)}/\rho_D\right]$, with mean rotational correlation time ρ_D = 0.35 ns and fundamental anisotropy r_0 = 0.375. The gray scale indicates an increasing number of single-molecule bursts from white to black. **c,** FRET histograms from MFD fitted to one donor-only state and two FRET states. **d,** TIRF time traces of surface-immobilized 3WJ; Alexa488 (green) and Cy5 (red) intensities, and calculated FRET efficiency (E_{FRET}) and idealized trace (red) from Hidden Markov modeling. **e,** FRET histograms from TIRF fitted to two FRET states. **f,** Transition density plot of TIRF FRET trace. Measured at 20 °C in a buffer containing 1 mM $MgCl_2$.

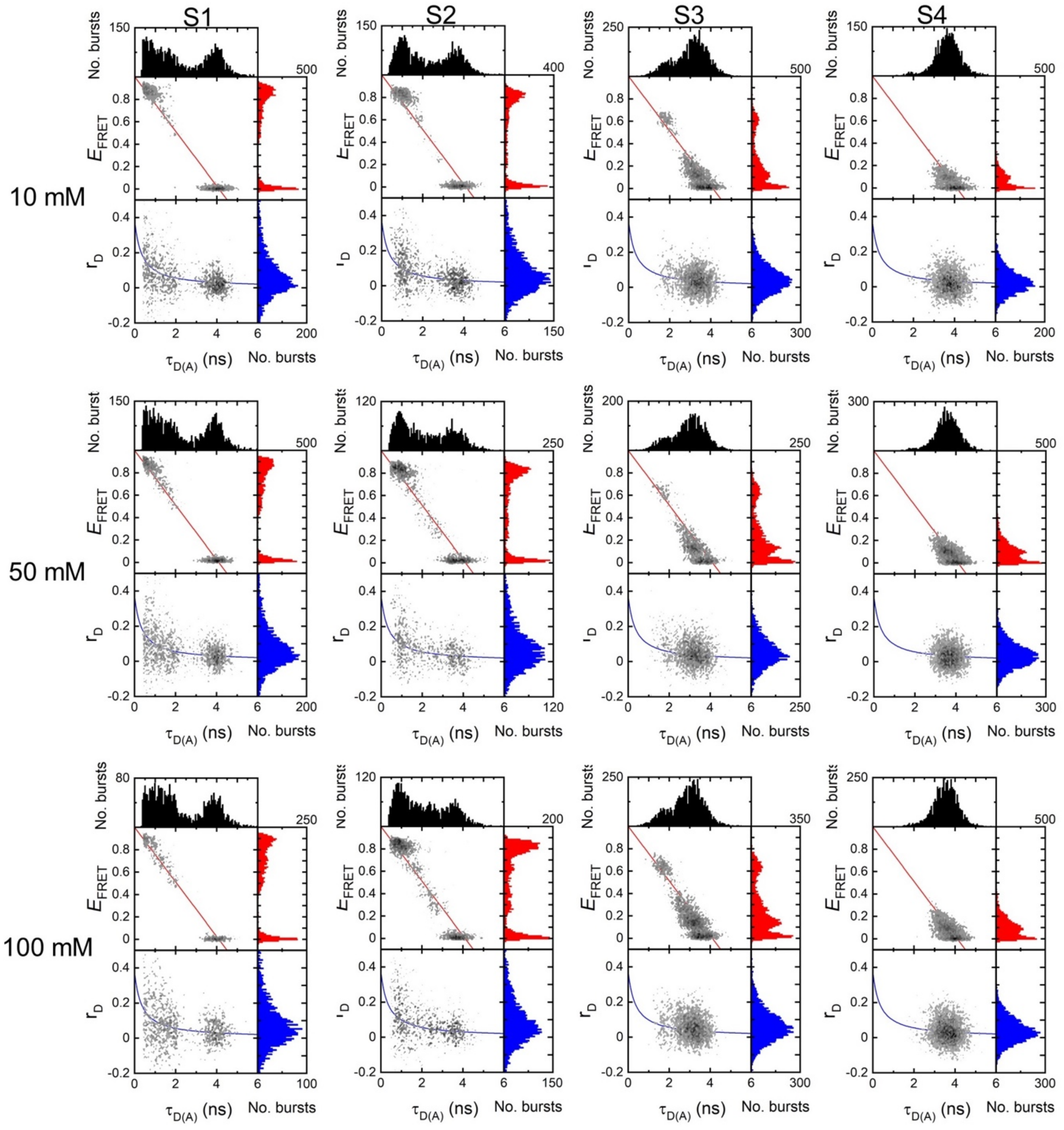

Supplementary Fig. 10. SM-FRET of static 3WJs using MFD vs. [Mg^{2+}]: 2D plots. 2D plot shows FRET efficiency (E_{FRET}) or donor anisotropy (r_D) vs donor lifetime [$\tau_{D(A)}$] for S1-4 (left to right) at $MgCl_2$ concentrations of 10 (top), 50 (middle), and 100 mM (bottom). The overlaid red line is the theoretical FRET relationship $E_{FRET} = 1 - [\tau_{D(A)}/\tau_D]$, where τ_D = 4.1 ns. The blue overlaid line is the Perrin equation $r_D = r_0/[1 + \tau_{D(A)}/\rho_D]$, with mean rotational correlation time ρ_D = 0.35 ns and fundamental anisotropy r_0 = 0.375. The gray scale indicates an increasing number of single-molecule bursts from white to black. The samples were measured at 20 ℃. See Supplementary Fig. 1 for the structures.

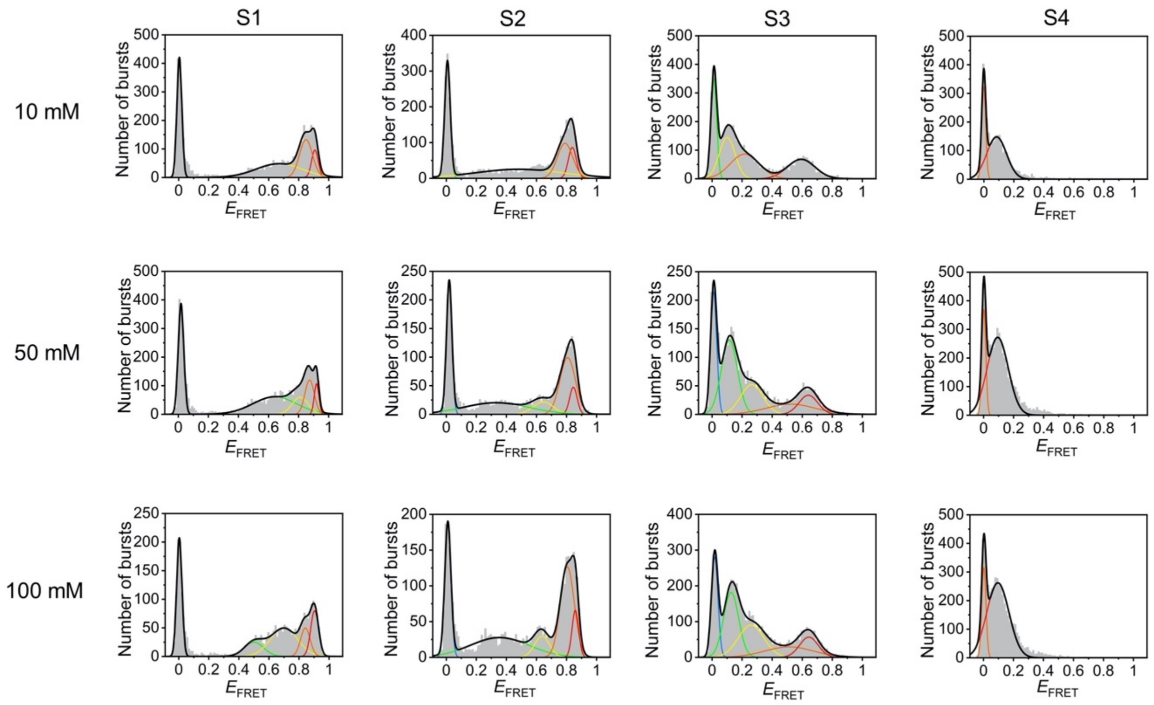

Supplementary Fig. 11. SM-FRET of static 3WJs using MFD vs. [Mg^{2+}]: FRET histograms. FRET histograms of FRET efficiency (E_{FRET}) vs. number of bursts for S1-4 (left to right) at $MgCl_2$ concentrations of 10 (top), 50 (middle), and 100 mM (bottom). Histograms for S4 were fitted to two Gaussians (for one donor-only and one FRET state). For S1-S3, four (10 mM) or five (50 and 100 mM) Gaussians were required, corresponding to three or four FRET states, respectively. Samples were measured at 20 ℃. See Supplementary Fig. 1 for the structures.

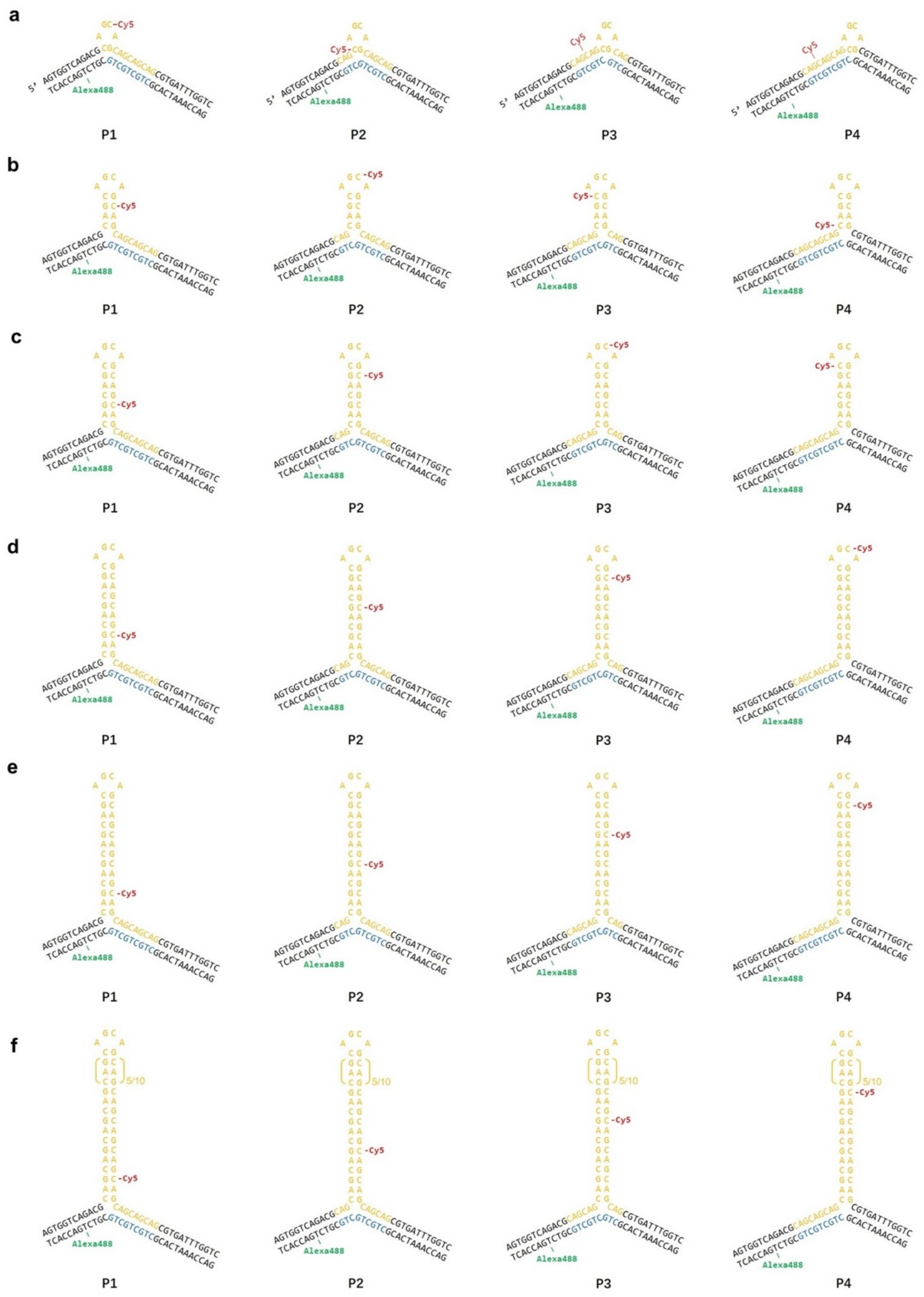

Supplementary Fig. 12. Structure of mobile 3WJs with $(CAG)_n$ slip-outs. a-f, Four positional isomers for n = 2 (**a**), n = 4 (**b**), n = 6 (**c**), n = 8 (**d**), n = 10 (**e**), n = 20/30 (**f**). For mobile 3WJs with $(CTG)_n$ slip-outs, CAG repeats in orange and the CTG repeats in blue are replaced by CTG and CAG repeats, respectively.

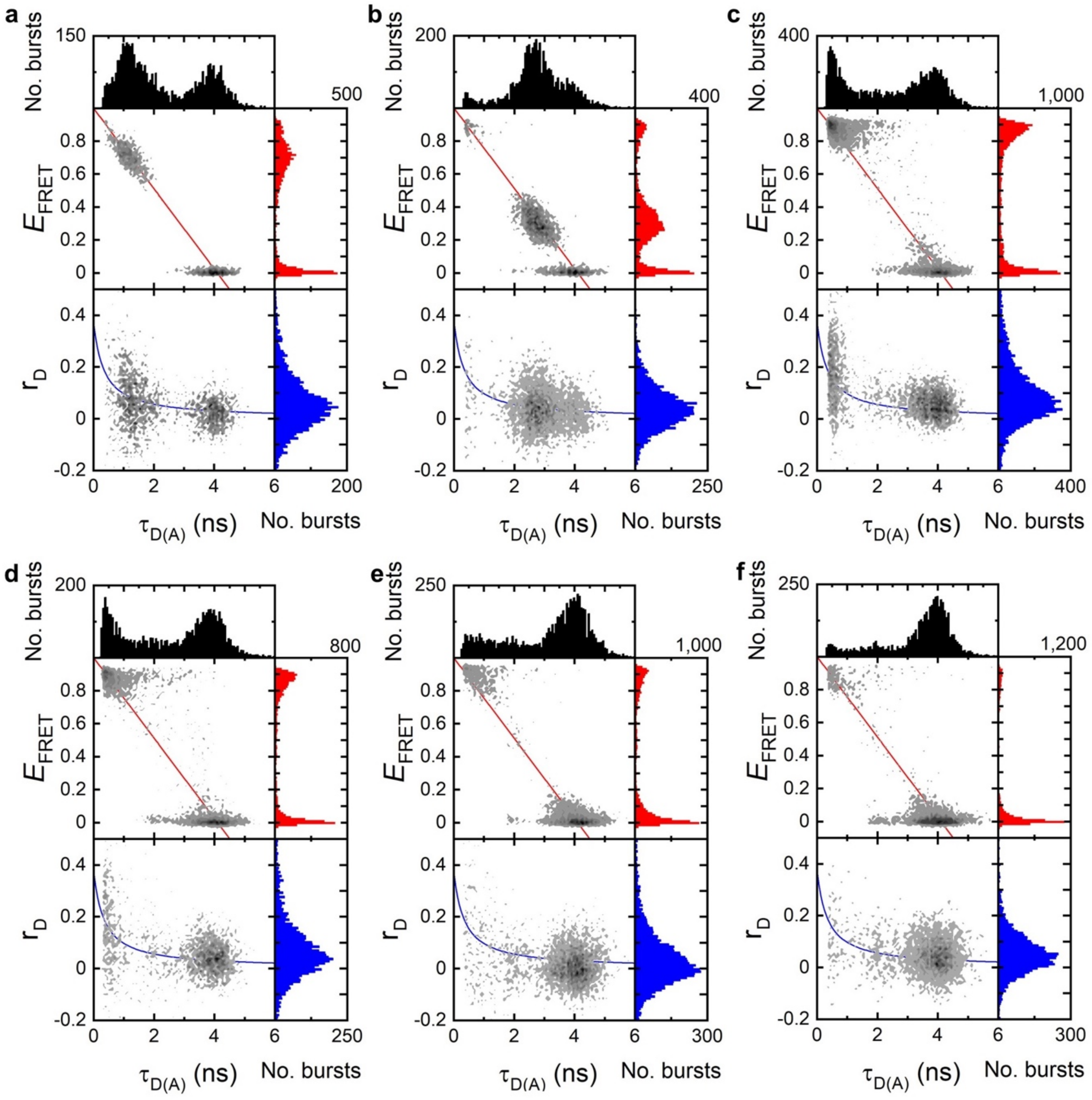

Supplementary Fig. 13. SM-FRET of mobile 3WJs with (CAG)$_n$ slip-out using MFD: 2D plots. a-f, 2D plot shows FRET efficiency (E_{FRET}) or donor anisotropy (r_D) vs donor lifetime [$\tau_{D(A)}$] for 3WJs with (CAG)$_n$ slip-out with n =2 (**a**), n=4 (**b**), n=6 (**c**) n=8 (**d**), n=20 (**e**) and n=30 (**f**). The overlaid red line is the theoretical FRET relationship $E_{FRET} = 1 - [\tau_{D(A)}/\tau_D]$, where τ_D = 4.1 ns. The blue overlaid line is the Perrin equation $r_D = r_0/[1 + \tau_{D(A)}/\rho_D]$, with mean rotational correlation time ρ_D = 0.35 ns and fundamental anisotropy r_0 = 0.375. The gray scale indicates an increasing number of single-molecule bursts from white to black. The samples were measured at 20 °C in a buffer with 1 mM $MgCl_2$.

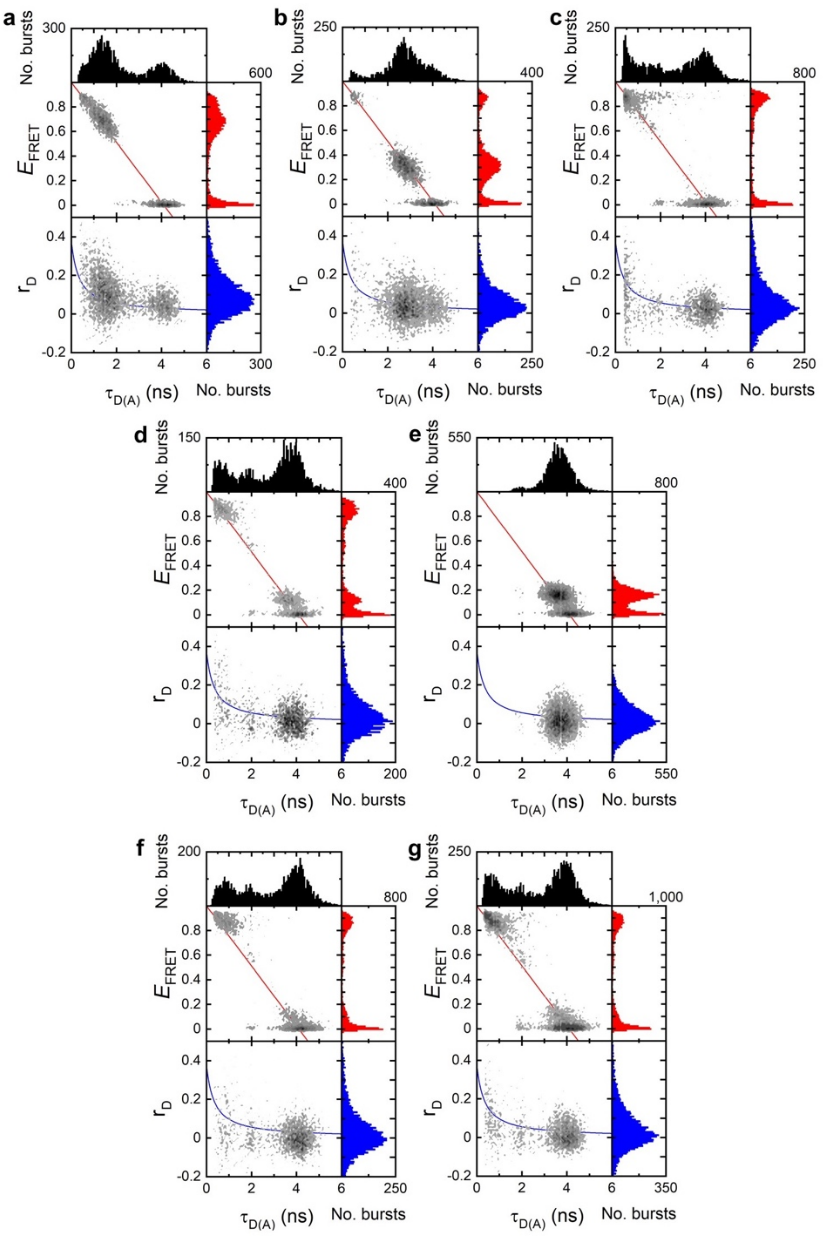

Supplementary Fig. 14. SM-FRET of mobile 3WJs with $(CTG)_n$ slip-out using MFD: 2D plots. a-g, 2D plot shows FRET efficiency (E_{FRET}) or donor anisotropy (r_D) vs donor lifetime [$\tau_{D(A)}$] for 3WJs with $(CTG)_n$ slip-out with n =2 (**a**), n=4 (**b**), n=6 (**c**) n=8 (**d**), n=10 (**e**), n=20 (**f**) and n=30 (**g**). The overlaid red line is the theoretical FRET relationship $E_{FRET} = 1 - [\tau_{D(A)}/\tau_D]$, where τ_D = 4.1 ns. The blue overlaid line is the Perrin equation $r_D = r_0/[1 + \tau_{D(A)}/\rho_D]$, with mean rotational correlation time ρ_D = 0.35 ns and fundamental anisotropy r_0 = 0.375. The gray scale indicates an increasing number of single-molecule bursts from white to black. The samples were measured at 20 °C in a buffer with 1 mM $MgCl_2$.

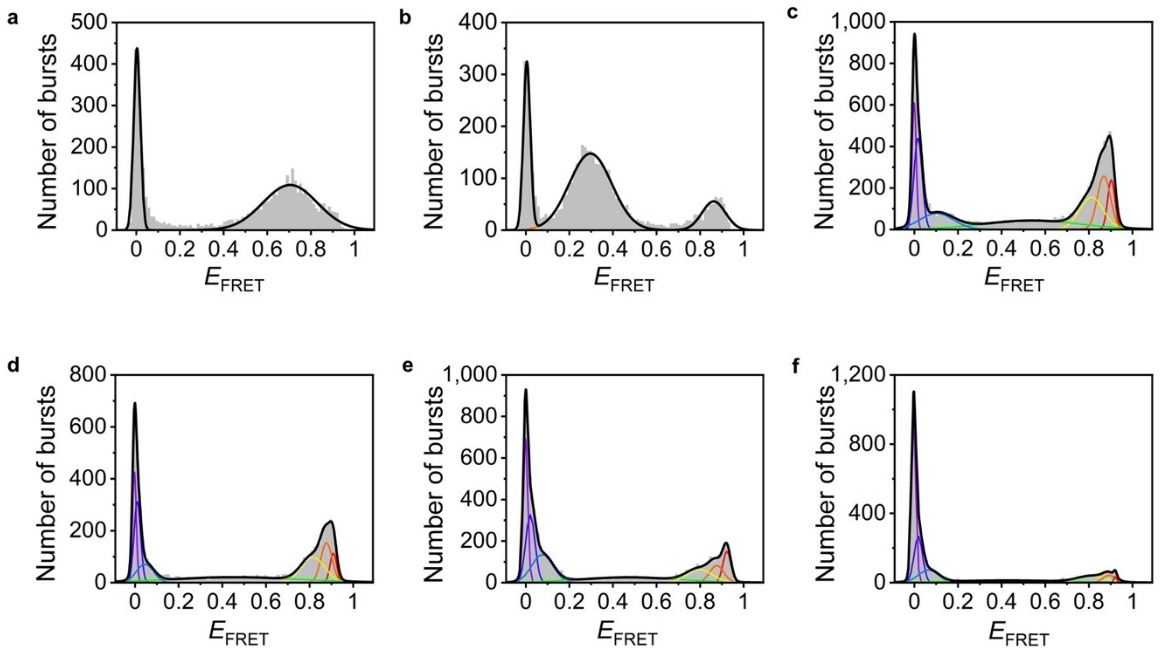

Supplementary Fig. 15. SM-FRET using MFD for (CAG)$_n$ mobile 3WJs: FRET histograms. FRET histograms for n = 2 (**a**), n = 4 (**b**) n = 6 (**c**) n = 8 (**d**), n = 20 (**e**), n = 30 (**f**) fitted to one (n=2), two (n = 4) or six (n = 6, 8, 20 and 30) FRET states. The samples were measured at 20 ℃ in a buffer with 1 mM $MgCl_2$. See Supplementary Fig. 12 for the structures.

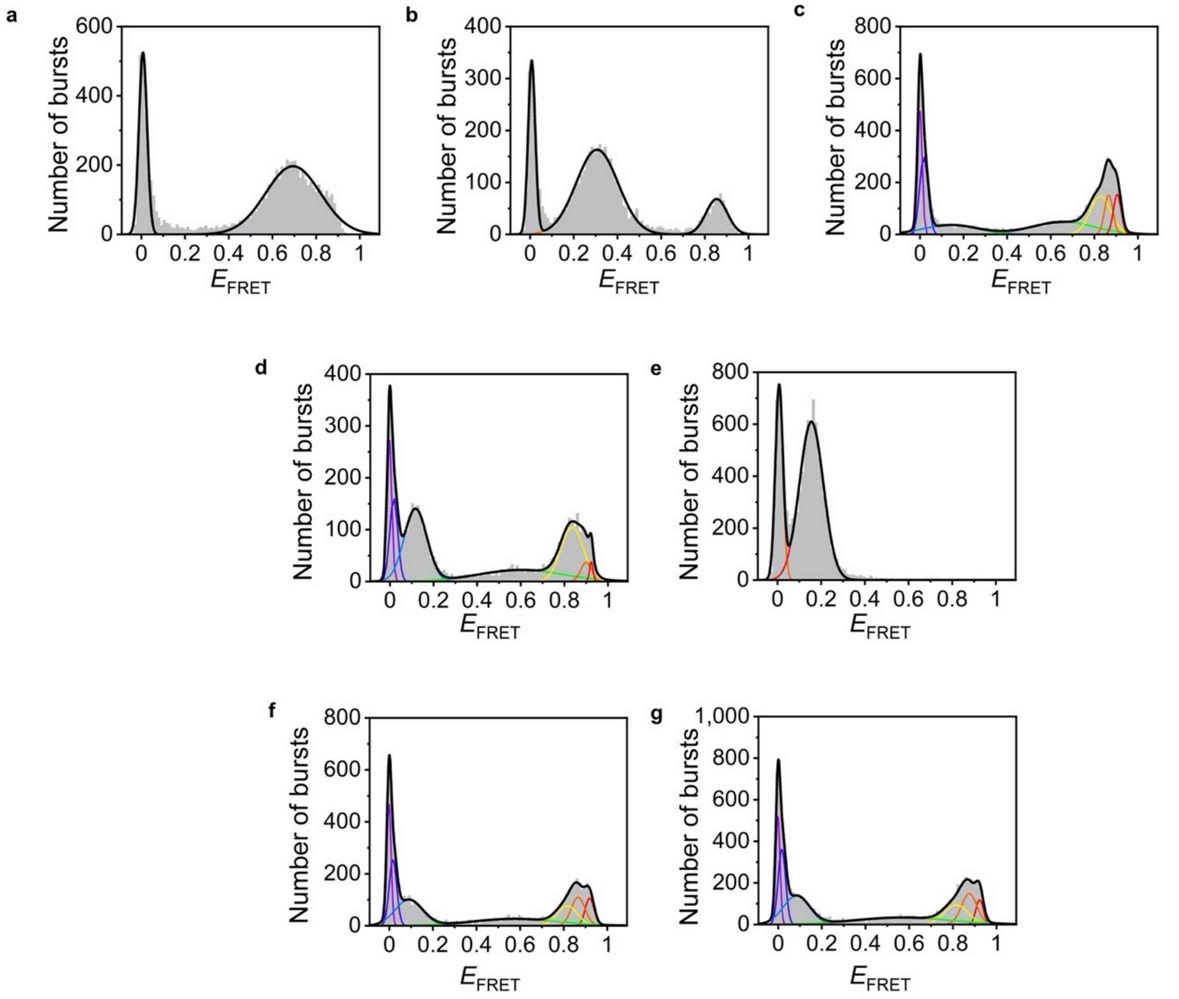

Supplementary Fig. 16. SM-FRET using MFD for $(CTG)_n$ mobile 3WJs: FRET histograms. FRET histograms for n = 2 (**a**), n = 4 (**b**) n = 6 (**c**) n = 8 (**d**), n = 10, (**e**) n = 20 (**f**), n = 30 (**g**) fitted to one (n=2, 10), two (n = 4) or six (n = 6, 8, 20 and 30) FRET states. The samples were measured at 20 ℃ in a buffer with 1 mM $MgCl_2$. See Supplementary Fig. 12 for the structures.

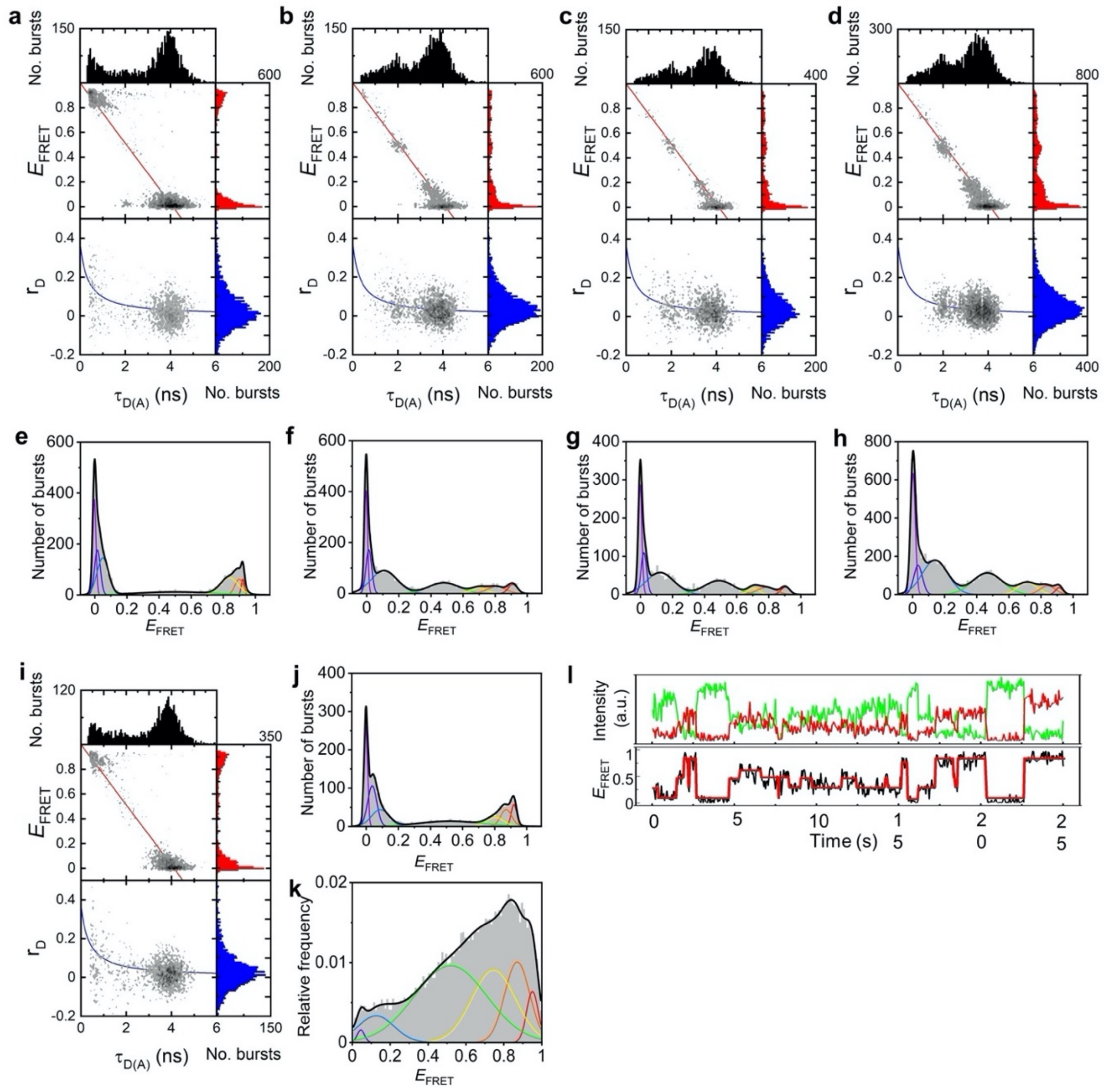

Supplementary Fig. 17. SM-FRET of mobile 3WJs with $(CAG)_{10}$ slip-out using MFD and TIRF as a function of Mg^{2+} concentration and at 37 °C. a-d and **i,** 2D plot of FRET efficiency (E_{FRET}) or donor anisotropy (r_D) vs donor lifetime [$\tau_{D(A)}$]. The overlaid red line is the theoretical FRET relationship $E_{FRET} = 1 - [\tau_{D(A)}/\tau_D]$, where τ_D = 4.1 ns. The blue overlaid line is the Perrin equation $r_D = r_0/[1 + \tau_{D(A)}/\rho_D]$, with mean rotational correlation time ρ_D = 0.35 ns and fundamental anisotropy r_0 = 0.375. The gray scale indicates an increasing number of single-molecule bursts from white to black. **e-h,** and **j,** FRET histograms from MFD fitted to one donor-only state and six FRET states. $MgCl_2$ concentrations are 0 mM (**a**, **e**), 1 mM (**i**, **j**) 10 mM (**b**, **f**), 50 mM (**c**, **g**), 100 mM (**d**, **h**). Samples were measured at 20 °C (**a-h**) or 37 °C (**i**, **j**). **k-l**, TIRF with 0 mM $MgCl_2$, 20 °C, showing the Alexa488 (green) and Cy5 (red) intensities, and calculated FRET efficiency (E_{FRET}) and idealized trace (red) from Hidden Markov modeling (**l**) and the corresponding FRET histogram (**k**).

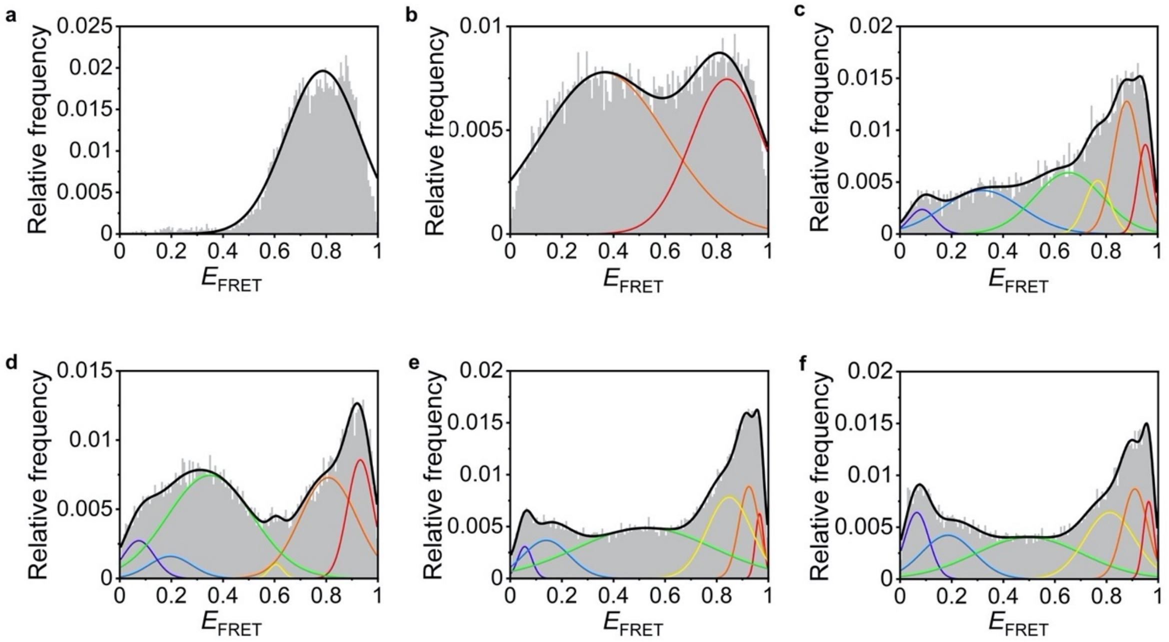

Supplementary Fig. 18. SM-FRET using TIRF for $(CAG)_n$ mobile 3WJs. FRET histograms for n = 2 (**a**), n = 4 (**b**) n = 6 (**c**) n = 8 (**d**), n = 20 (**e**), n = 30 (**f**), fitted to one (n=2), two (n = 4) or six (n = 6, 8, 20 and 30) Gaussians. The samples were measured at 20 ℃ in a buffer with 1 mM $MgCl_2$. See Supplementary Fig. 12 for the structures.

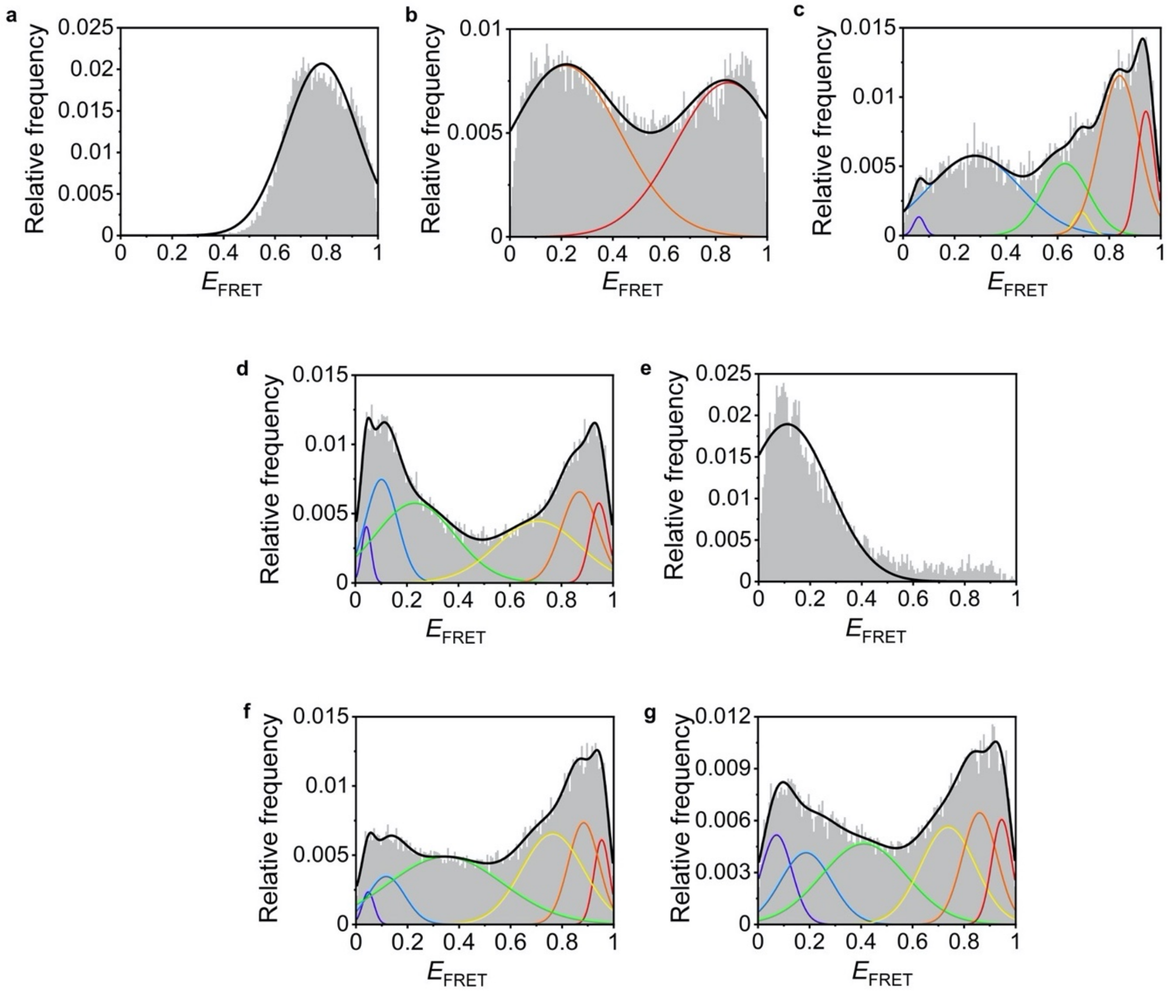

Supplementary Fig. 19. SM-FRET using TIRF for $(CTG)_n$ mobile 3WJs. FRET histograms for n = 2 (**a**), n = 4 (**b**) n = 6 (**c**) n = 8 (**d**), n = 10 (**e**), n = 20 (**f**), n = 30 (**g**) fitted to one (n=2, 10), two (n = 4) or six (n = 6, 8, 20 and 30) Gaussians. The samples were measured at 20 ℃ in a buffer with 1 mM $MgCl_2$. See Supplementary Fig. 12 for the structures.

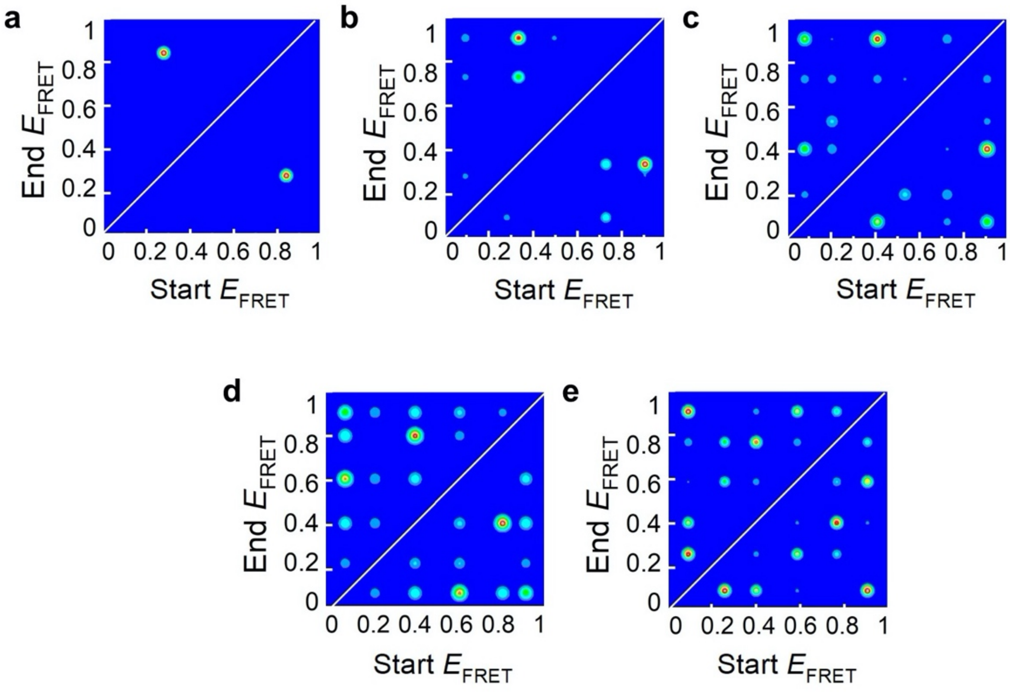

Supplementary Fig. 20. Dependence of dynamics on repeat length. Transition density plots from TIRF time traces of 3WJs with three complementary CAG and CTG repeats in the duplex and $(CTG)_n$ slip-outs (n = 4, 6, 8, 20 and 30). **a,** n = 4. **b,** n = 6. **c,** n = 8. d**,** n = 20. e**,** n = 30. Measured at 20 °C in a buffer containing 1 mM $MgCl_2$.

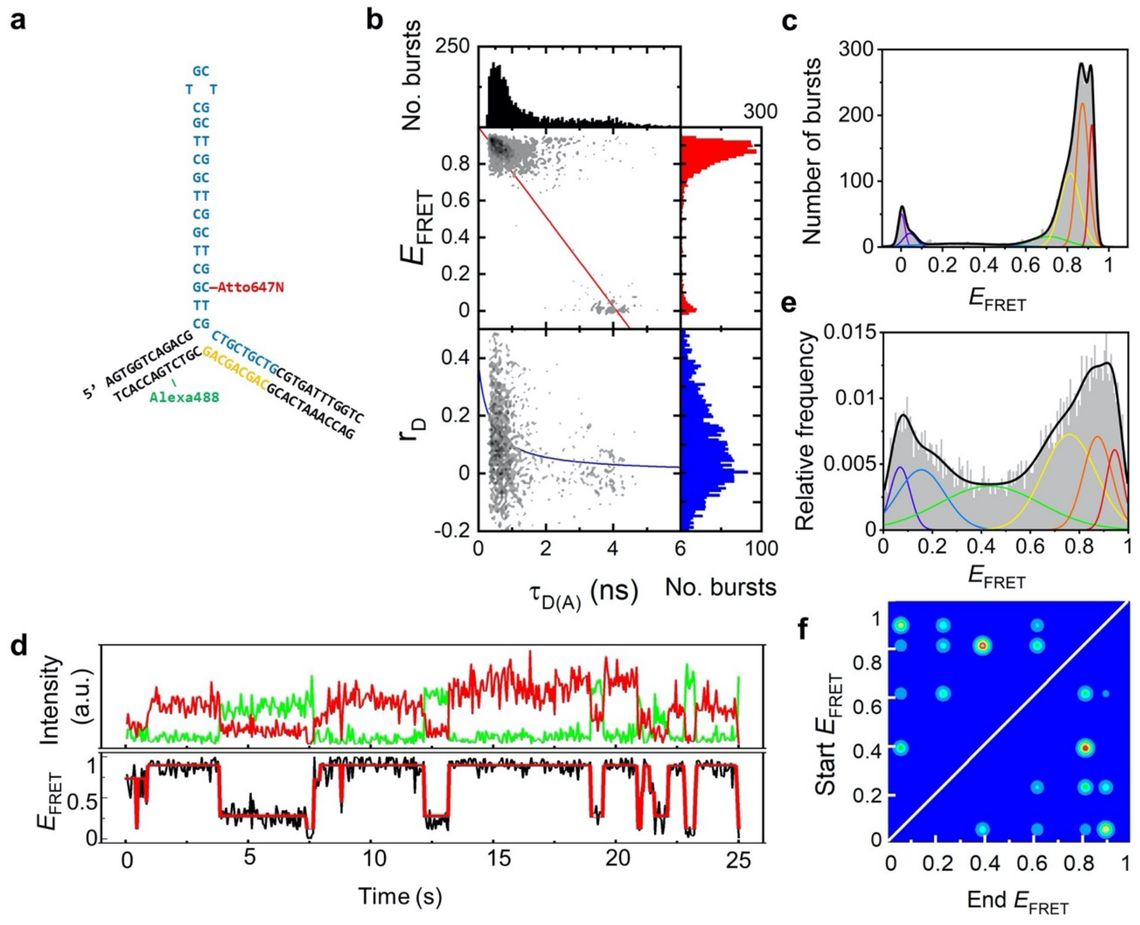

Supplementary Fig. 21. SM-FRET for a mobile 3WJ with $(CTG)_{10}$ slip-out labelled with Atto647N. a, Structure of the 3WJ (corresponding to positional isomer P1 in Supplementary Fig. 12e). **b,** 2D plot shows FRET efficiency (E_{FRET}) or donor anisotropy (r_D) vs donor lifetime [$\tau_{D(A)}$]. The overlaid red line is the theoretical FRET relationship $E_{FRET} = 1 - [\tau_{D(A)}/\tau_D]$, where τ_D = 4.1 ns. The blue overlaid line is the Perrin equation $r_D = r_0/[1 + \tau_{D(A)}/\rho_D]$, with mean rotational correlation time ρ_D = 0.35 ns and fundamental anisotropy r_0 = 0.375. The gray scale indicates an increasing number of single-molecule bursts from white to black. **c,** FRET histograms from MFD fitted to one donor-only state and six FRET states. **d,** TIRF time traces of surface-immobilized 3WJ; Alexa488 (green) and Cy5 (red) intensities, and calculated FRET efficiency (E_{FRET}) and idealized trace (red) from Hidden Markov modeling. **e,** FRET histograms from TIRF fitted to six FRET states. **f,** Transition density plots of TIRF FRET traces. Measured at 20 °C in a buffer containing 1 mM $MgCl_2$.

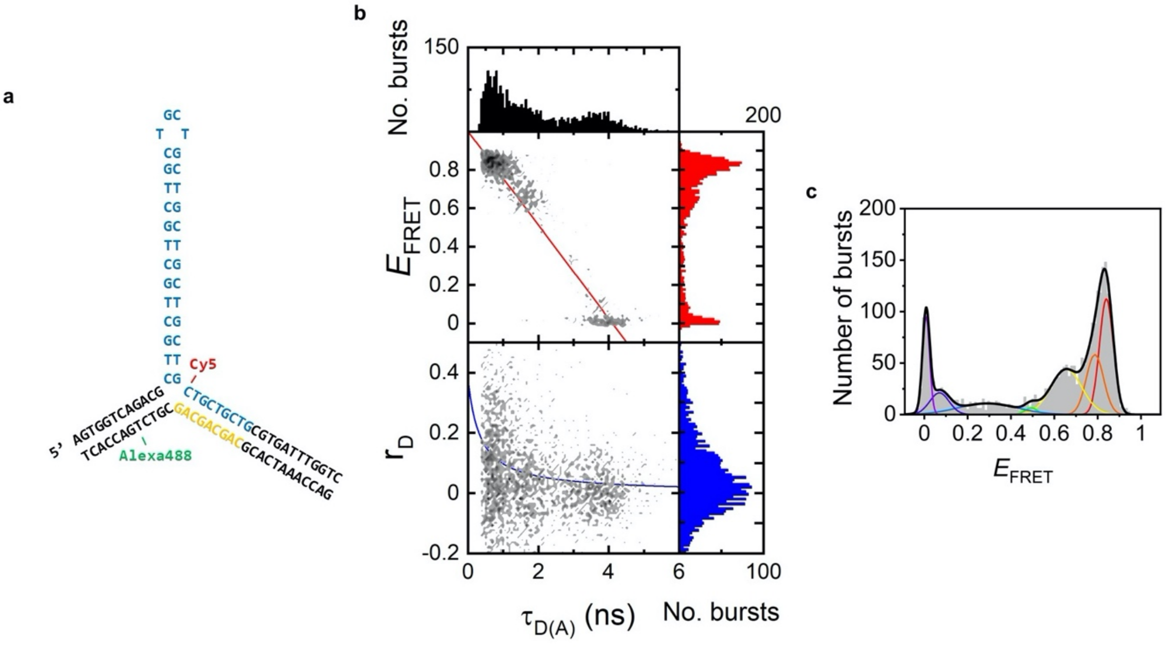

Supplementary Fig. 22. SM-FRET for a mobile 3WJ with $(CTG)_{10}$ slip-out labelled with Cy5 in an alternate position. a, Structure of the 3WJ (corresponding to positional isomer P1 in Supplementary Fig. 12e). **b,** FRET efficiency (E_{FRET}) or donor anisotropy (r_D) vs donor lifetime [$\tau_{D(A)}$]. The overlaid red line is the theoretical FRET relationship $E_{FRET} = 1 - [\tau_{D(A)}/\tau_D]$, where τ_D = 4.1 ns. The blue overlaid line is the Perrin equation $r_D = r_0/[1 + \tau_{D(A)}/\rho_D]$, with mean rotational correlation time ρ_D = 0.35 ns and fundamental anisotropy r_0 = 0.375. The gray scale indicates an increasing number of single-molecule bursts from white to black. **c,** FRET histograms from MFD fitted to one donor-only state and six FRET states. Measured at 20 °C in a buffer containing 1 mM $MgCl_2$.
